# Modeling and Piezoelectric Analysis of Nano Energy Harvesters

**DOI:** 10.3390/s20143931

**Published:** 2020-07-15

**Authors:** Muhammad Faisal Wasim, Shahzadi Tayyaba, Muhammad Waseem Ashraf, Zubair Ahmad

**Affiliations:** 1 Department of Physics (Electronics), GC University, Lahore 54000, Pakistan; rfwasim@yahoo.com; 2 Department of Computer Engineering, The University of Lahore, Lahore 54000, Pakistan; Shahzadi.Tayyaba@hotmail.com; 3 Center for Advanced Material (CAM), Qatar University, PO Box 2713, Doha, Qatar

**Keywords:** aluminum, ANSYS, piezoelectric, MEMS, ZnO nanorods

## Abstract

The expedient way for the development of microelectromechanical systems (MEMS) based devices are based on two key steps. First, perform the simulation for the optimization of various parameters by using different simulation tools that lead to cost reduction. Second, develop the devices with accurate fabrication steps using optimized parameters. Here, authors have performed a piezoelectric analysis of an array of zinc oxide (ZnO) nanostructures that have been created on both sides of aluminum sheets. Various quantities like swerve, stress, strain, electric flux, energy distribution, and electric potential have been studied during the piezo analysis. Then actual controlled growth of ZnO nanorods (NRs) arrays was done on both sides of the etched aluminum rod at low-temperature using the chemical bath deposition (CBD) method for the development of a MEMS energy harvester. Micro creaks on the substrate acted as an alternative to the seed layer. The testing was performed by applying ambient range force on the nanostructure. It was found that the voltage range on topside was 0.59 to 0.62 mV, and the bottom side was 0.52 to 0.55 mV. These kinds of devices are useful in low power micro-devices, nanoelectromechanical systems, and smart wearable systems.

## 1. Introduction

Sensing and energy production are the hot issues of the modern era, along with price control of these technologies. However, various types of mechanical energy in the ambient base physical environment such as typing stress, walking and running, vehicles, noise, and other sounds and other industrial vibrations can be collected to be used in electronic devices [1]. Different forms of mechanical energy that are found in a natural environment like the flow of water, blowing of wind, and tide still need to be explored [2]. Microelectromechanical system (MEMS) based devices exhibit modern features. Smart materials play an important role in creating multifunctional characteristics in these devices. Nanostructures have changed the functionality and dimensions of these devices. Most of these devices are based on the nanostructure basic functionality that are part of modern energy devices. Nanostructures of different dimensions are used in different MEMS devices to perform various tasks such as sensing, actuation, balancing, controlling, and pumping [3]. To achieve these functionalities, one dimensional (1D) nanostructures like nanorods, nanowires, nanotubes, nano-tapes, and nano-belts can play a vital role [4,5,6,7]. Nanorods, nanotubes, and thin film like structures are extensively used for energy harvesting devices. The piezoelectric and other physical aspects of the nanomaterials have also been used to manufacture the MEMS devices in the nano-regime for energy generation [8]. Zinc oxide is most suitable material for synthesis of 1D nanostructures by using various techniques such as electron beam gun evaporation, thermal vacuum evaporation (TVE), metal-organic chemical vapor deposition (MOCVD), molecular beam epitaxial (MBE) growth, sputtering, chemical bath deposition (CBD), dip coating, and hydrothermal methods [9]. Vertical highly aligned, uniform, and straight arrays of the ZnO nanorods on surfaces and their interfaces have a prominent role in the performance of MEMS devices. ZnO nanorods aligned in the c-axis of applied stress can cause deformation to develop an electric field [10]. The vibrational energy harvester with a piezoelectric effect was reported with a downshift of resonance frequencies [11]. Dynamic vibrational effects are important and can be studied with a bi-stable electromagnetic harvester [12]. These piezoelectric energy harvesters can be tuned to various methods, like the biasing flip method [13]. The degree of freedom (DOF) is a critical parameter in the dynamic mode of harvesters [14]. Piezoelectric and electromagnetic effects are also useful for energy harvesters based on wind effects [15]. Such vibrational piezoelectric material based MEMS energy harvesters are crucial for self-power electronic devices with micropower, nano electromechanical systems, and smart wearable systems [1]. 

In this work, the authors have adopted a low-temperature CBD method for the growth of ZnO nanostructures due to its simplicity and low-cost. The etched aluminum substrate has been used for fabrication of the nanorods. ANSYS software was used for piezo simulation. The testing of the developed MEMS energy harvester was also performed. 

## 2. Design, Working Principle, and Dependent Parameters of Nano-Harvesters

The piezoelectric analysis comprises coupling the electric field and structure. When functional force is applied on nanorods, the piezoelectric material exhibits compression, and contrariwise the vibrations create voltage. The direct effect of piezoelectricity can be represented by the general equation given below

(1) D=dT+ℰE

where,

$D=Electrical\;Polarization\;\left(\frac{C}{m^{2}}\right)$

$T=Stress\;Vector\;(\frac{N}{m^{2}}$)

d=Piezoelectric Coefficent matrix

ℰ=Electrical Permitivity matrix

*E* = *Electic Field vector* ($\frac{V}{m}$)

**Figure 1:**
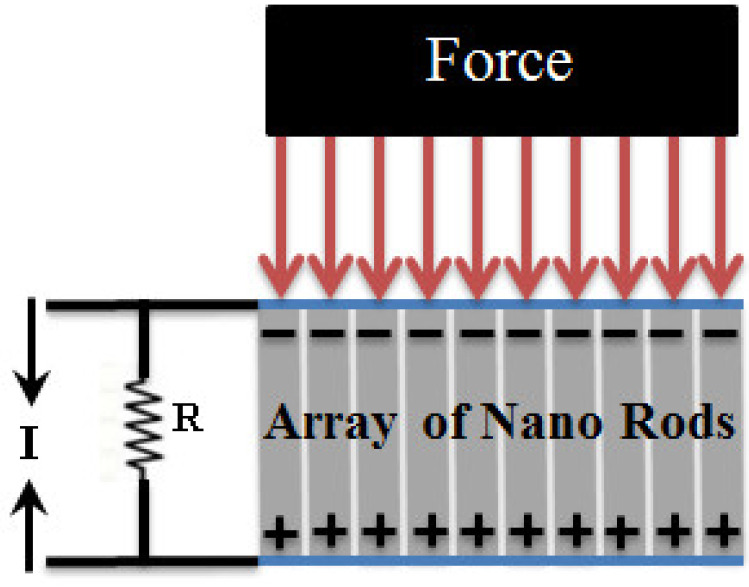
Schematic of nano-generator working principle.

The electric field is applied parallel to the z-axis. The direction of positive polarization is customarily parallel with the *z-*axis, and conversely, the strain is applied in the x-axis. The schematic of the nano-generator working principle is shown in Figure 1.

**Figure 2:**
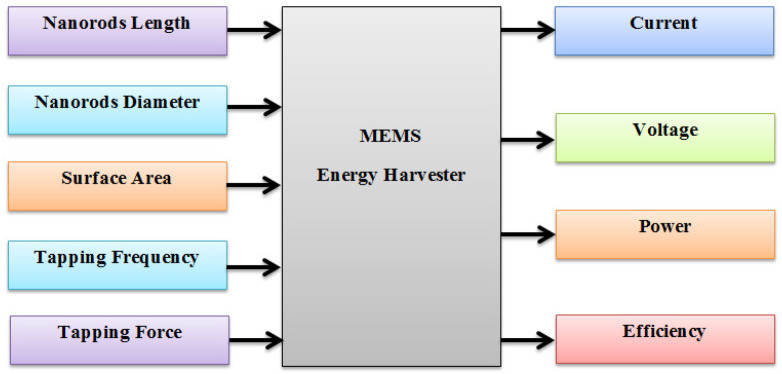
Schematic of parametric dependency of nano-generators.

The performance and robustness of the nano-generator depend on various parameters. Some of these parameters are involved during synthesis or fabrication stages, and rests are involved in the harvesting stage. The fabrication stage parameters are the nanorod length, diameter, and the surface area of the device. The harvesting stage parameters are tapping force and frequency. The current, voltage, power, and efficiency of nanogenerators directly depend on the parameters of the above two stages. The schematic of parametric dependency is shown in Figure 2. 

## 3. ANSYS Simulation

**Figure 3:**
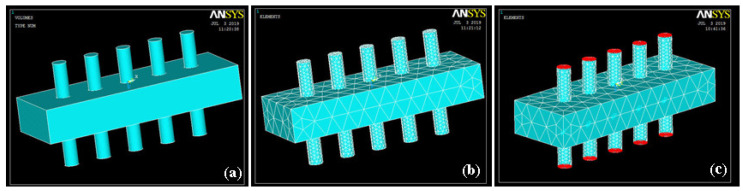
Geometry of the (**a**) solid; (**b**) mesh; (**c**) load.

ANSYS (ANSYS 17 perpetual license purchased by Ibadat Education Trust, the University of Lahore, Pakistan) was used for the simulation of dual side array of ZnO nanorods. This array was created on aluminum sheets/rods. After creating the 3D geometry of the nanorod array, the material properties of ZnO were defined. Then, structural element Solid 226 and circuit element Circuit 94 were chosen, and meshing of aluminum substrate with a glued array of rods was performed. Required boundary conditions, such as degree of freedom, and loads, such as force, were applied to the model. The 3D geometry, mesh model, and model with load are shown in Figure 3. 

**Figure 4:**
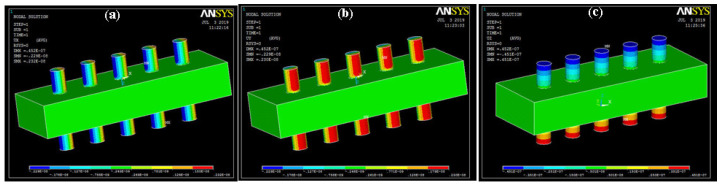
Deflection along direction (**a**) X; (**b**) Y; (**c**) Z.

The simulation was carried out in APDL, and results were visualized and analyzed [16]. As the structural and piezo elements were defined during simulation, bending, stress, strain, electrical potential, energy distribution, and electrical flux were observed. The deflection of 3D geometry at the applied loads occurred in the X, Y, and Z directions, as shown in Figure 4. 

**Figure 5:**
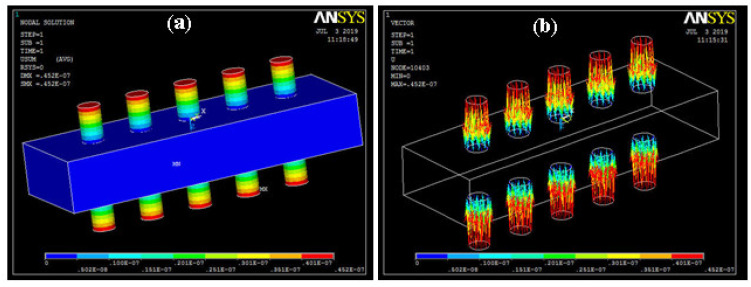
Total deformation (**a**) contour plot; (**b**) vector form.

The deflection of the presented 3D model can be calculated along all directions. The contour and vector plot of the veer are shown in Figure 5. It was found that the maximum value in the bar was 4.52 µm.

**Figure 6:**
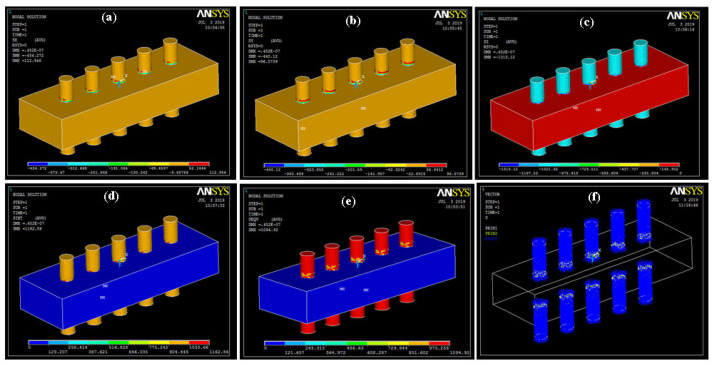
Stress (**a**) X; (**b**) Y; (**c**) Z; (**d**) intensity; (**e**) Von Mises; (**f**) vector form.

Due to the uniform applied load on the geometry, the stress occurs in the X, Y, and Z directions and can be analyzed. Stress-related values are shown in Figure 6. The values of maximum stress intensity and Von Mises stress were 1162.86 and 1094.92 Pa, respectively. 

**Figure 7:**
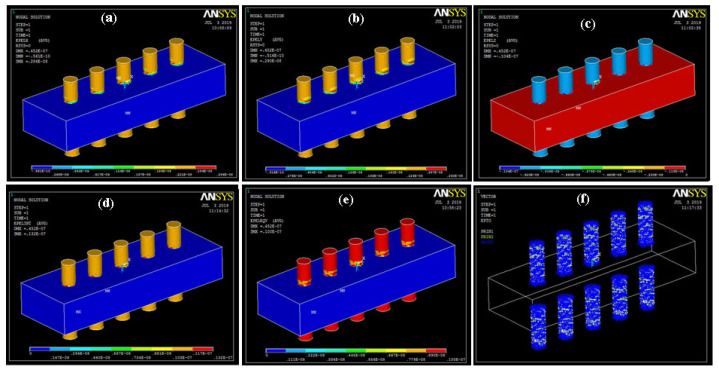
Strain (**a**) X; (**b**) Y; (**c**) Z; (**d**) intensity; (**e**) Von Mises; (**f**) vector form.

Change in geometry occurs due to stress, and its values in the X, Y, and Z directions are shown in Figure 7. The maximum values of strain intensity and Von Mises strain were 13.2 × 10^−9^ and 10 × 10^−9^. During the piezoelectric analysis, it was found that the ZnO rod array was stable, and the values related to stress were in the limit of the elastic range. 

**Figure 8:**
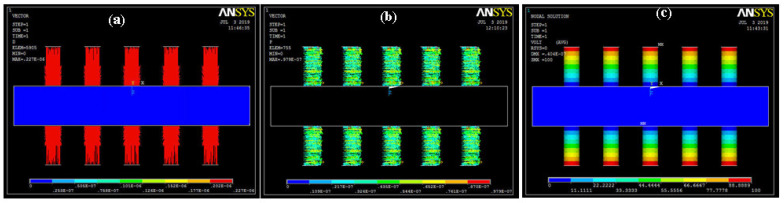
Piezoelectric quantities: (**a**) electrical flux density; (**b**) energy distribution; (**c**) electrical potential distribution.

As the arrays of ZnO rods were on both sides of an aluminum sheet, piezo properties were defined and analyzed on only the rod-like structure. The value of voltage was considered 0–100 V during the piezo analysis. Therefore, the electrical potential distribution was 0–100 V, as given in the Figure 8. This distribution can be visualized on both sides of the nanorods during the applied load due to the polarity difference. Electrical flux, energy distribution, and electrical potential are shown in Figure 8. The maximum values of electrical flux, energy distribution, and electrical potential are 227 × 10^−9^ c/m^2^, 97.9 × 10^−9^ watts, and 100 V, respectively. 

## 4. Manufacturing

**Figure 9:**
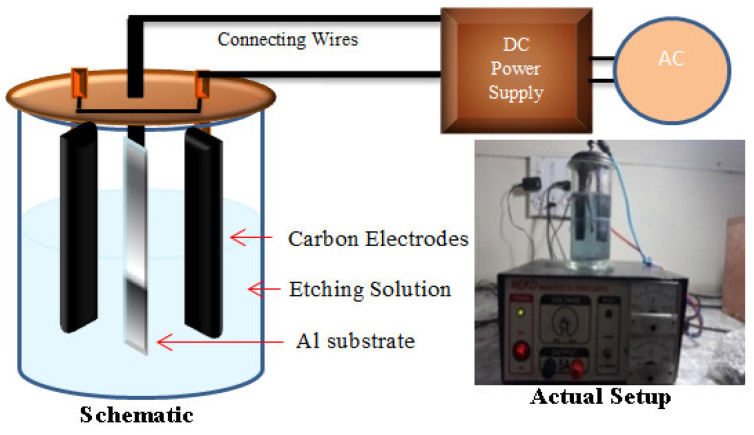
Carbon parallel plates chemical etching setup.

The main steps involved in experimentation were cleaning, etching, growth on the nanostructure, development of a connection, and testing. The development and testing of the energy harvester can be performed using the technique which was reported in our previous work [1]. First, the pure and smooth aluminum sheet of 0.5 mm thick was cut into a piece of the size 2 cm × 5 cm. Then, cleaning of the aluminum substrate was performed using the standard method. Then, etching was carried out [17]. The solution was prepared by adding 60 g sodium chloride (NaCl) into 500 mL of deionized water. This solution was put into the self-developed etching setup. The etching vessel consisted of two flat carbon bars separated by a small distance and adjusted parallel to each other. The aluminum substrate was fixed between these carbon-based parallel electrodes. The voltage of 30 V was applied for 10 min. Then the substrate was washed and dried in an oven. The actual and schematic diagram of the etching setup is shown in Figure 9. 

**Figure 10:**
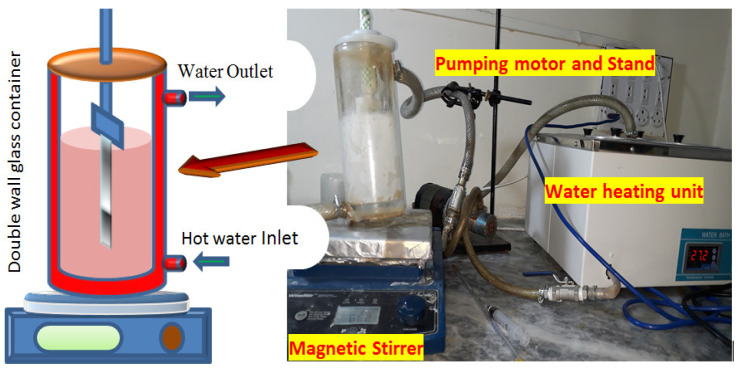
Chemical bath deposition schematic and functional diagram.

After etching the aluminum substrate, the ZnO nanorods were deposited using chemical bath deposition. This method provides a constant temperature inside the chemical bath for the uniform growth of nanorods. The etched substrate was treated with dodicanthiol for 3 h and then dried at 80 °C. This treated substrate was attached with a Teflon catcher and inserted into the beaker at constant magnetic stirring. The beaker contained a 15 mM solution of zinc acetate di-hydrate and hexa-amine. The temperature of the beaker was 95 °C. The position of the substrate was flipped every 30 min and the solution was changed after 2 h. This process continued for 8 h. Then the substrate was annealed at 400 °C. The schematic and actual CBD setup is shown in Figure 10.

## 5. Characterization and Testing

**Figure 11:**
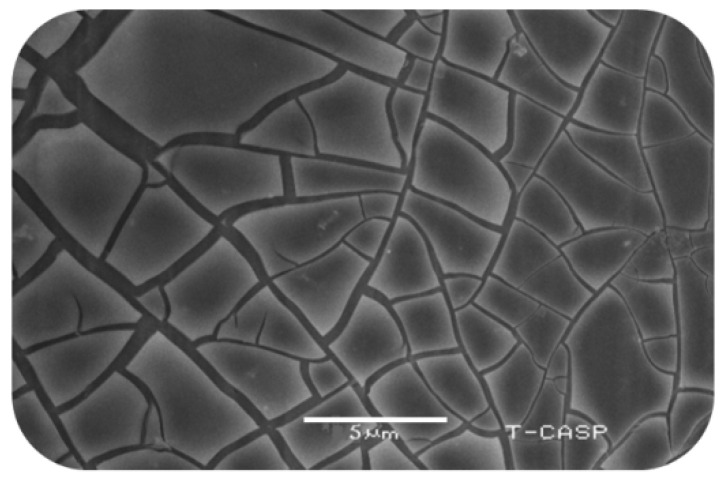
SEM graph of the etched aluminum substrate.

The etched sheet of aluminum was viewed using a scanning electron microscope (SEM) and the image is shown in Figure 11. It is clear that small creaks appeared on the surface. These micro creaks have been used as an alternative to the seed layer. Normally, the seed layer aligns and gives strength to nanorods. 

**Figure 12:**
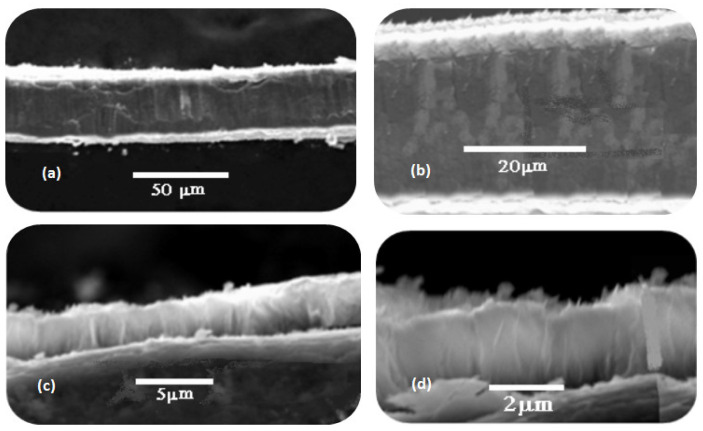
SEM cross-sectional images of ZnO nanorods: (**a,b**) side view; (**c**) top edge view; (**d**) bottom edge view.

The cross-sectional view of the nanorods on the etched aluminum substrate sheet is shown in Figure 12. It was found that the rod length was around 4 µm, and the width of the used aluminum thick sheet was determined to be 50 µm (0.05 mm). The morphological picture clarifies the vertical alignment of the synthesized nanorods. 

**Figure 13:**
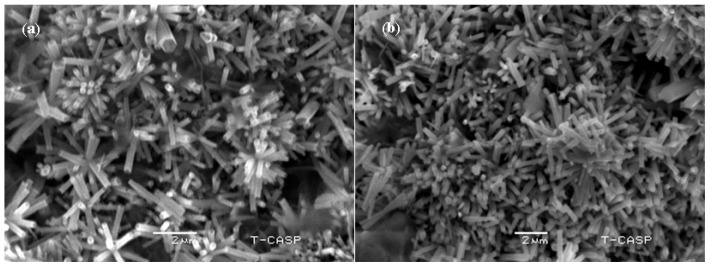
Surface view of SEM images of ZnO nanorods: (**a**) topside view; (**b**) bottom side view.

The sample surface was also viewed by SEM, as shown in Figure 13. These nanorods were vertically aligned and highly dense. 

**Figure 14:**
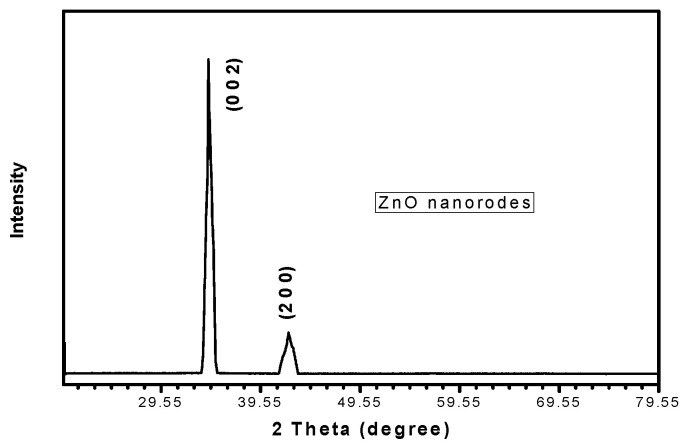
XRD patterns.

Figure 14 shows the X-ray diffraction (XRD) patterns of the ZnO nanorods grown on the etched aluminum sheet. A hexagonal wurtzite structure has been found with space group P63mc, (ICDD card no: 04-008-8198), which is the typical structure of ZnO. The sharpness of the (0 0 2) XRD peaks indicated the growth of the ZnO nanorods in the vertical direction, which were the most important for the piezoelectric effect. No peaks of any new phases were detected in the XRD patterns, which show the uniformity of the synthesized nanorods. 

**Figure 15:**
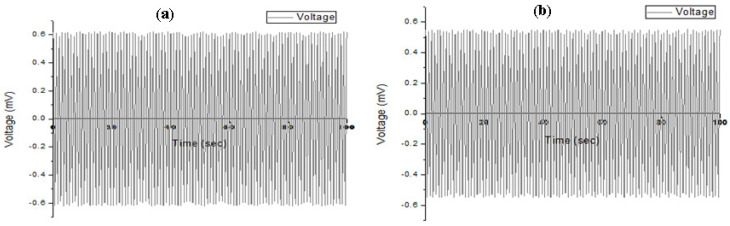
Open circuit voltage: (**a**) top side; (**b**) bottom side.

After the synthesis of ZnO nanostructures, the MEMS energy harvester was developed by making contacts using a supporting technique. The open-circuit voltage was studied. To measure the output parameters of the piezoelectric generators, the required frequency, known as the mechanical resonance frequency, gives the optimized harvest power. But, the conditions for the vibrational excitation frequencies are different from the resonance frequency. The power of generation can be improved by using impedance matching and adjusting the load. To accomplish such a condition the load circuit and impedance of the generator were matched to get the maximum power. An energy harvesting circuit was used for impedance matching. The cyclic force was applied on the generator using test setup to harvest the energy that setup consisted of the cam follower mechanism. The voltage observed on the topside was in the range of 0.59 to 0.62 mV, and the bottom side was 0.52 to 0.55 mV, as shown in Figure 15.

Generally, the outputs of the ZnO based MEMS energy harvester such as current, voltage, power, and efficiency depend on factors like nanorod length, nanorod diameter, surface area or sample size, tapping frequency, and tapping force. Harvesting conditions are also important for the MEMS energy harvester, along with impedance matching. There is no output power if the resonant frequency does not match with exciting frequency. Hence, more power can be achieved by load matching with impedances [18,19]. Various researchers reported a single sided nano-generator with the different substrates [20,21] while we have developed a nano-generator on both sides of the substrate by growing the piezo material on both sides of the substrate. Khan et al. [22] reported a similar device that was fabricated on nanoporous aluminum oxide with a large sample size with single side piezo material while Kasi et al. reported an electro polishing system to fabricate such a membrane [23]. Their growth time was 5 hr, while the current study shows the nanostructure growth in 4 h. Nanomaterial based MEMS energy harvesters can be fabricated using chemical and physics deposition methods. Reproducibility is one of the challenging aspects of using chemical methods. The physical methods have more accuracy than chemical methods due to the optimized parameters. On the other hand, chemical methods are low cost and easy to fabricate with less percentage of reproducibility. Sufficient energy harvesting power is required to operate medical, microfluidics, and small-scale power devices [24,25,26]. By using directional piezoelectric energy, the harvester is more suitable for such applications [27]. The new design presented in this study with two side growth of nano piezo-material for energy harvesting can also be helpful to fulfill the requirements of small scale devices.

## 6. Conclusions

The optimization of various parameters by using soft computing techniques is a modern method. This study presents the simulation and development of MEMS-based energy harvesters. The fabrication of the harvester was reported using a cost-effective method. First, the piezo analysis for the array of nanorods on both sides of the aluminum substrate was performed to optimize different qualities such as veer, stress, strain, energy distribution, electric flux, and electric potential. Then, micro cracks were developed on both sides of the substrate, acting as an alternative to the seed layer. The seed layer gives the strength and alignment to nanorods. The synthesis of zinc oxide nanorods was performed on both sides of aluminum sheet at low temperature using the CBD method for the development of a MEMS energy harvester. After making contact, the open circuit voltage was measured using a cam follower testing setup by adjusting the impedance matching. It was found that the voltage range on the topside was 0.59 to 0.62 mV and the bottom side was 0.52 to 0.55 mV as observed during testing. It was concluded that such devices are useful in self-powered electronic devices with microwatt power, nano electromechanical systems, and smart wearable systems.
